# Quantitative Evaluation of Intensity Inhomogeneity Correction Methods for Structural MR Brain Images

**DOI:** 10.1007/s12021-015-9277-2

**Published:** 2015-08-26

**Authors:** Marco Ganzetti, Nicole Wenderoth, Dante Mantini

**Affiliations:** Neural Control of Movement Laboratory, ETH Zurich, 8057 Zurich, Switzerland; Department of Experimental Psychology, University of Oxford, Oxford, OX1 3UD UK; Laboratory of Movement Control and Neuroplasticity, KU Leuven, 3001 Leuven, Belgium

**Keywords:** Bias field, Brain structure, Comparative study, Intensity non-uniformity, Magnetic resonance imaging

## Abstract

The correction of intensity non-uniformity (INU) in magnetic resonance (MR) images is extremely important to ensure both within-subject and across-subject reliability. Here we tackled the problem of objectively comparing INU correction techniques for T1-weighted images, which are the most commonly used in structural brain imaging. We focused our investigations on the methods integrated in widely used software packages for MR data analysis: FreeSurfer, BrainVoyager, SPM and FSL. We used simulated data to assess the INU fields reconstructed by those methods for controlled inhomogeneity magnitudes and noise levels. For each method, we evaluated a wide range of input parameters and defined an enhanced configuration associated with best reconstruction performance. By comparing enhanced and default configurations, we found that the former often provide much more accurate results. Accordingly, we used enhanced configurations for a more objective comparison between methods. For different levels of INU magnitude and noise, SPM and FSL, which integrate INU correction with brain segmentation, generally outperformed FreeSurfer and BrainVoyager, whose methods are exclusively dedicated to INU correction. Nonetheless, accurate INU field reconstructions can be obtained with FreeSurfer on images with low noise and with BrainVoyager for slow and smooth inhomogeneity profiles. Our study may prove helpful for an accurate selection of the INU correction method to be used based on the characteristics of actual MR data.

**Electronic supplementary material:**

The online version of this article (doi:10.1007/s12021-015-9277-2) contains supplementary material, which is available to authorized users.

## Introduction

Magnetic Resonance Imaging (MRI) is a valuable technique for studying the structural properties of the human brain. Due to its non-invasive nature, significant imaging contrast, high spatial resolution, and reasonable acquisition times, it is largely used to investigate alterations in brain structure associated with neurodegenerative and neuropsychiatric disorders (Braga et al. 2012; Chen et al. 2011; Duncan et al. 2013; Frisoni et al. 2010; Tillema and Pirko 2013). A serious issue in the analysis of MR structural images is, however, the reproducibility of the imaging results, both within and across subjects, that arises from smooth intensity variations across the whole MR image (Belaroussi et al. 2006; Bernstein et al. 2006). These variations can be referred to as *intensity non*-*uniformity* (*INU*), but also *intensity inhomogeneity* or *spatial bias*. The magnitude and spatial profile of the INU may be influenced by several factors, among which static field inhomogeneity, reduced radio frequency coil uniformity, radio frequency (RF) penetration, gradient-driven eddy currents, inhomogeneous reception sensitivity profile, and overall subject anatomy and position (Belaroussi et al. 2006; Mihara et al. 1998; Simmons et al. 1994; Sled and Pike 1998; Vovk et al. 2007). The acquisition pulse sequence and the field strength play also an important role in determining the INU (Belaroussi et al. 2006; Bernstein et al. 2006; Boyes et al. 2008). In particular, an imperfect spatial homogeneity of the static magnetic field B_0_ is the main cause of slow intensity variations across the imaged volume when the MR field strength is relatively low. At higher MR field strengths the contribution of B_0_ will diminish as the other effects, as for example tissue-dependent distortions produced by MR gradients, will start to become much more significant and not behave in a manner that suits the INU correction methods assumptions (Bernstein et al. 2006; Moser et al. 2012; Umutlu et al. 2014).

To correct the INU in MR structural images, a number of prospective calibration methods were proposed (Belaroussi et al. 2006; Vovk et al. 2007). These are generally intended to account for hardware-related factors that hamper MR image quality. For instance, it was suggested that the INU may be compensated by acquiring supplementary images of uniform phantoms (Axel et al. 1987), combining information from different coils (Brey and Narayana 1988; Murakami et al. 1996), merging data obtained from multiple datasets (Liney et al. 1998), and designing dedicated imaging sequences (Deichmann et al. 2002; Mihara et al. 1998), Nevertheless, it is important to note that those methods can eliminate hardware-related but not subject-induced inhomogeneities. Furthermore, the usefulness of prospective approaches is narrowed by the need of dedicated acquisitions, the limited stability and the sensitivity to input parameters (Belaroussi et al. 2006; Likar et al. 2001; Vovk et al. 2007).

Given the limitations of prospective methods, retrospective INU correction methods, which rely only on image features to remove spatial inhomogeneities, are nowadays more widely used. Notably, they can be applied to structural images with different features, and can theoretically account for both hardware-related and subject-induced INU components (Hou 2006; Vovk et al. 2007). Many retrospective INU correction methods have been proposed in the last years. In spite of their different implementations, a common characteristic is that they model the INU field as a spatial function describing slowly changing intensity variations across the volume. This INU field is typically assumed to be multiplicative, in that the intensity of the inhomogeneity is proportional to that of the INU-free MR image at the same location (Ashburner and Friston 2005; Axel et al. 1987; Belaroussi et al. 2006; Vovk et al. 2007). Also, the presence of an additive hardware-related noise should be taken into account when dealing with actual structural MR images. Notably, performance of an INU correction method can be influenced both by INU characteristics and by noise in various ways, depending on its specific features and implementation.

Since an effective INU correction is critical for investigations of brain structure, previous studies have attempted to compare the performance of several approaches. A number of comparative studies on retrospective INU correction methods were conducted (Arnold et al. 2001; Likar et al. 2001; Velthuizen et al. 1998; Vovk et al. 2006), but none of them recently. This leaves an unanswered question on whether and to what extent newly developed methods outperform older ones. Furthermore, previous comparative studies focused exclusively on default parameters for each INU correction method. Nonetheless, since each method performs better or worse depending on the specific settings used, the selection of optimal parameters is becoming an important topic in MRI (Boyes et al. 2008; Uwano et al. 2014; Weiskopf et al. 2011; Zheng et al. 2009).

In this study we conduct a quantitative assessment of INU correction methods for T1-weighted images, which are the most commonly used images to investigate brain structure. Specifically, we focus on the methods implemented in the most recent versions of Statistical Parametric Mapping (www.fil.ion.ucl.ac.uk/spm), FMRIB Software Library (www.fmrib.ox.ac.uk/fsl), FreeSurfer (www.freesurfer.net) and BrainVoyager (www.brainvoyager.com), respectively. We use simulated data to compare the method results with a ground truth at different INU field magnitudes and image noise levels. Furthermore, we examine a wide range of input parameters for each method, so that we can define their enhanced configuration and compare its performance with those obtained using default input parameters.

## Methods

### INU Correction: Theory and Algorithms

In this section, we first introduce the theoretical background for modeling of the INU effects on MR images, and then we describe how each method attempts to remove it from the data.

#### Modelling of MR Intensity Inhomogeneities

According to the RF field mapping theory (Insko and Bolinger 1993; Stollberger and Wach 1996), intensity inhomogeneities can be modeled as multiplicative. The majority of the studies (Arnold et al. 2001; Belaroussi et al. 2006; Dawant et al. 1993; Pham and Prince 1999; Wells et al. 1996) suggested that the corruption of MR images by intensity inhomogeneity can be formalized as follows:

1 $$ u\left(x,y,x\right)=v\left(x,y,z\right)\cdot b\left(x,y,z\right)+n\left(x,y,z\right) $$

where *u*(*x*, *y*, *x*) is the actual image and *v*(*x*, *y*, *z*) is the INU-free noiseless image, *b*(*x*, *y*, *z*) is the INU field and *n*(*x*, *y*, *z*) is additive spatial noise. While the INU field is slowly varying, the noise has high spatial frequency and its values show a Rician distribution (Andersen 1996; Belaroussi et al. 2006; Gudbjartsson and Patz 1995; Henkelman 1985). Intensity inhomogeneity in MR images can be corrected by estimating the INU field *b*(*x*, *y*, *z*). This, indeed, permits to approximate the INU-free image *v*(*x*, *y*, *x*) starting from the actual image *u*(*x*, *y*, *x*). It is worth noting that the contribution of the noise, due to its high spatial frequency, cannot be eliminated by means of the INU correction (Vovk et al. 2007). To accomplish the INU correction, different methods have been developed in the last years. We will focus hereafter on methods implemented in widely used MR imaging software packages.

#### Methods Under Investigation

##### Statistical Parametric Mapping (SPM)

The INU correction in SPM12 (RRID: nif-0000-00343) is implemented within the unified segmentation module (Ashburner and Friston 2005). It is indeed integrated with brain segmentation, as this allows the joint optimization of both analysis steps. Specifically, since intensity inhomogeneity is detrimental for the image segmentation process, the INU correction is iteratively performed until convergence of the segmentation results. This is achieved combining a Finite Gaussian Mixture (FGM) model with a deformable template (tissue probability atlas). In this respect, a mixture of Gaussians is used to model the intensity distribution from different tissue types. By default, SPM uses more than one Gaussian for each tissue, since tissue probability maps may be shared across different classes (partial volume effects). On the other hand, built-in (prior) probability maps of different tissues are registered to the subject image. Afterwards, Bayesian statistics rules are used to calculate posterior probabilities that combine the template information with that contained in brain tissues. The INU field correction algorithm of SPM models smooth intensity variations by a linear combination of discrete cosine transform (DCT) basis functions. In other words, SPM represents the MR image as a sum of sinusoids of varying magnitude and frequency (basis functions). Due to the low-frequency nature of image inhomogeneities, slowly varying INU fields are isolated in terms of DCT components below a certain cut-off threshold.

An effective segmentation, and therefore an effective INU correction, is based on the minimization of the objective function derived from the FGM model along with the deformable template. The fitting of the model (i.e. minimization of the objective function) is performed following an Iterated Conditional Modes (ICM) approach. In other words, each iteration involves an estimate of different groups of parameters, while holding others at their optimal current solution. Upon convergence, the toolbox provides structural images that are INU-corrected and segmented.

##### FMRIB Software Library (FSL)

As for SPM, the INU field correction method implemented in FSL v5.0 (RRID: nif-0000-00305) is integrated with the segmentation tool, called FMRIB’s Automated Segmentation Tool (FAST). The FSL method estimates the INU field by fusing information from a FGM model and from a Hidden Markov Random Field (HMRF) model (Zhang et al. 2001). The FGM model is used as in SPM to decompose the image histogram into a mixture of Gaussians. On the other hand, the HMRF model considers the image information to be encoded through contextual constraints of neighboring voxels, so that the presence of noise, strong INU fields, and mainly partial volume effects can be taken into account. The objective function to be minimized is derived from a combination of FGM and HMRF, and is optimized using an ICM approach. Model parameters are estimated by means of an expectation maximization (EM) approach in the framework of image segmentation, iterating between tissue classification and intensity inhomogeneity correction. The expectation step consists of computing the maximum a posteriori estimate (MAP) of the INU field and the tissue labels. In turn, the maximization step is accomplished by computing the maximum likelihood estimate of the model parameters using the INU field and the tissue labels of the expectation step. As mentioned above, the MAP principle is employed to obtain the optimal estimate of the INU field starting from the observed intensity values. By comparing the actual voxel intensities with the predicted ones, a residual field is calculated. Then, this residual field is low-pass filtered to obtain an estimate of the INU field. This procedure can be iterated multiple times. By estimating the EM solution, INU correction and brain segmentation can be performed at the same time.

##### FreeSurfer (FS)

FreeSurfer v5.3 (RRID: nif-0000-00304) includes a famous INU correction method developed by the Montreal Neurological Institute (MNI), and known as N3 (Sled et al. 1998). This method considers the intensity at each voxel as an independent distributed random variable. The basic assumption is that the INU field has a blurring effect on the MR image, reducing the high frequency components that characterize the image. As a result, the method tries to find the smooth INU field that maximizes the frequency content of the image intensity distribution. This is achieved through an iterative process that goes through three sequential steps: sharpening of the INU-corrupted intensity distribution, INU field estimate and INU field smoothing. The iterative process terminates when no significant changes in the estimated INU field are detected.

##### BrainVoyager (BV)

BrainVoyager QX (RRID: nif-0000-00274) implements an intensity inhomogeneity correction method based on a surface fitting approach. By means of low-order polynomials, the algorithm models low-frequency variations across the image (Dawant et al. 1993). The INU detection is accomplished using intensity information of voxels presumably located in the white matter. After brain extraction, the labelling of white mater reference points is achieved in two steps. The first phase is based on voxel intensity criteria. The idea behind that is that surface fitting is most reliable if it is estimated on voxels with higher intensity, and these are likely to be found in the white matter. However, this assumption may not hold in presence of heavy inhomogeneity profiles as well as low signal-to-noise ratio. To overcome this hindrance, a heuristic fully automated approach based on intensity information from neighbouring voxels to label as reference points in the white matter (Hou et al. 2006). Then, the intensities of reference voxels are fit by low-order polynomials following a least-squares approach. In this manner, it is possible to detect intensity variation across the whole volume. Finally, the INU field is generated using the calculated low-order polynomials, and is regressed out from the actual image. Optionally, the procedure of reference point labelling and INU field estimation may be iterated, aiming at minimizing residual errors. It is worth noting that the use of multiple iterations can introduce additional low-frequency noise in the estimated INU-free image if the residual INU to be estimated has very low magnitude and surface fitting becomes less reliable.

### Analysis of INU Correction Methods

In this section we describe how we built a realistic simulation to include spatial inhomogeneity in MR images, how the four INU correction methods were set up and tested, and how the performance of these methods were assessed.

#### Simulated Data

**Fig. 1:**
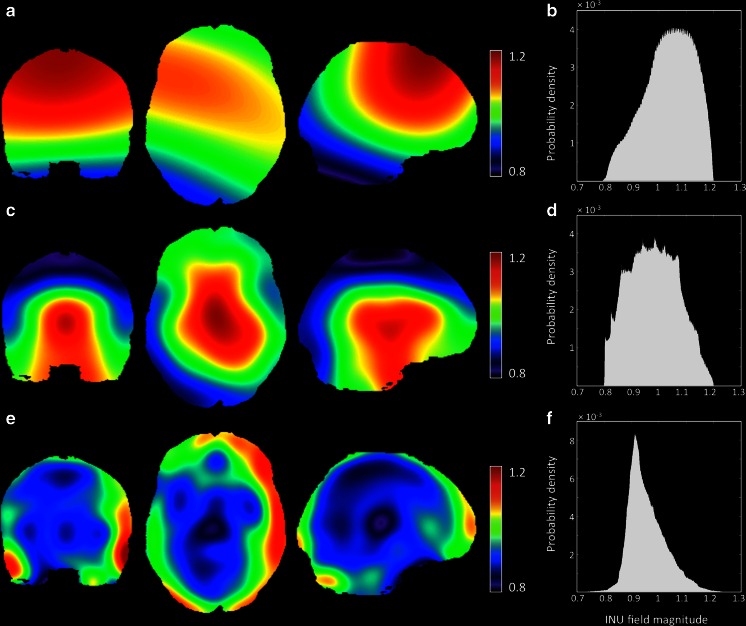
Simulated INU fields. The simulated INU fields at 40 % level are represented in coronal (*y* = 4), axial (*z* = 0), and sagittal (*x* = −11) sections for the BIAS 1.5 T (**a**), BIAS 3 T (**c**) and BIAS 7 T (**e**) profile. Histograms of the respective INU field are reported on the right side for the BIAS 1.5 T (**b**), BIAS 3 T (**d**) and BIAS 7 T (**f**) profile. It is worth noting that the INU field at 80 % level is characterized by the same spatial profile of the one at 40 % (range from 0.8 to 1.2), whereas the field values range from 0.6 to 1.4

A first simulated INU field was created using images generated from the BrainWeb MRI Simulator (RRID: nif-0000-00020, brainweb.bic.mni.mcgill.ca/brainweb). We first extracted a realistic INU field map (denoted as “field_A”) for the T1-w imaging modality, generated using known spatial varying perturbation of the RF pulse flip angle (Kwan et al. 1999). This field has slowly varying and smooth spatial profile, consistent with intensity inhomogeneities that are typically observed with 1.5 T MR scanners (Fig. 1a and b), and intensity values between 0.9 and 1.1 (corresponding to 20 % spatial variation). Then, we generated other two intensity inhomogeneity fields, which are intended to better reproduce inhomogeneities from 3 T (Fig. 1c and d) and 7 T (Fig. 1e and f) MR scanners, respectively. As proposed by Vovk and co-workers, the fields were created by cubic B-spline interpolation between equally spaced nodes at 60 and 40 mm in each direction (Vovk et al. 2004). These fields have an increased complexity in the spatial profile compared to the BrainWeb MRI Simulator field, reflecting intensity inhomogeneities that are typically generated by 3 and 7 T MR scanners, respectively. Intensity values for the two additional INU fields were initially set to have 20 % spatial variation. Thereafter, we generated fields also with 40, 60 and 80 % variation, by rescaling the image with 20 % variation to have values ranging between 0.8 and 1.2, 0.7 and 1.3, 0.6 and 1.4, respectively. For the sake of simplicity, we will refer to the three INU fields as BIAS 1.5 T, BIAS 3 T and BIAS 7 T, respectively.

**Fig. 2:**
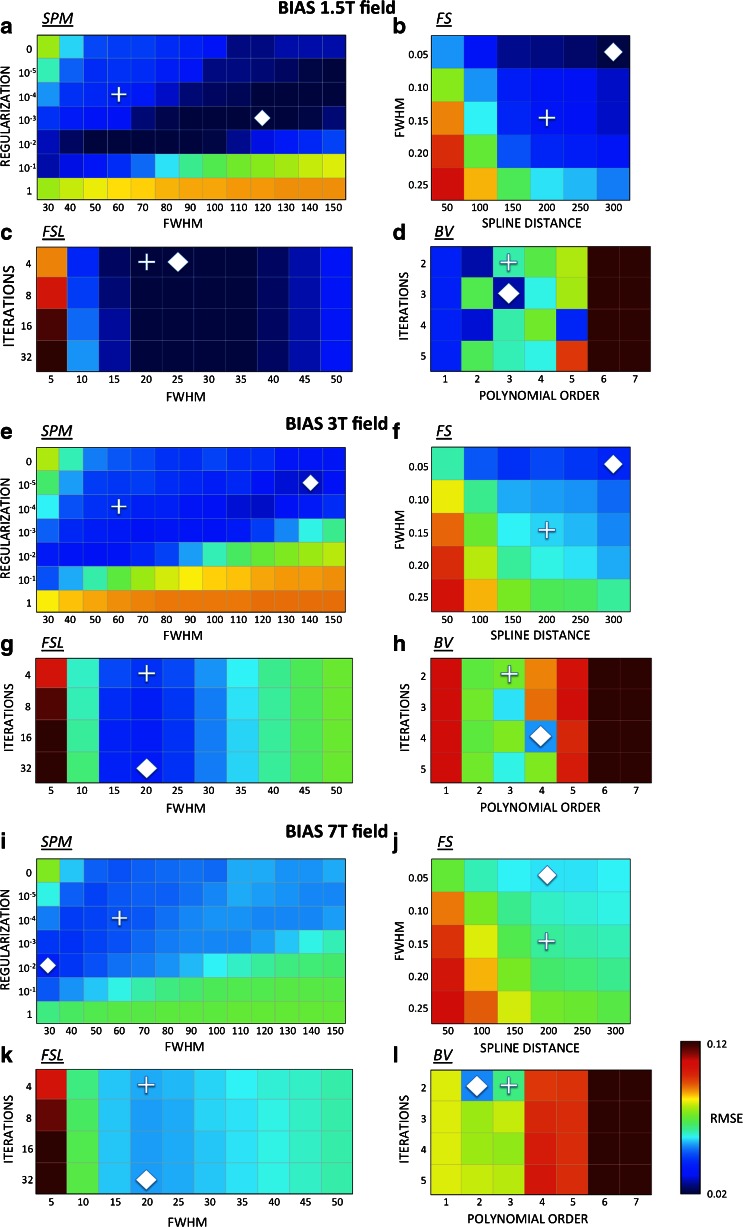
Dependence of INU field estimate on input parameters. In order to define the intensity inhomogeneity correction for each method and for each INU field (BIAS 1.5 T, BIAS 3 T and BIAS 7 T), we analysed several parameter configurations. We computed the RMSE between the simulated and the estimated INU field for each configuration. The default (indicated with a cross marker) and the enhanced configuration (indicated with a diamond marker) are shown for SPM (**a**, **e**, **i**), FS (**b**, **f**, **j**), FSL (**c**, **g**, **k**) and BV (**d**, **h**, **l**). In SPM, the *regularization* and the *bias field smoothing* (*FWHM*) parameters were varied. The *deconvolution kernel* (*FWHM*) and the *spline smoothing distance* parameters were varied in FS. In contrast, the *number of iterations* and the *bias field smoothing* (*FWHM*) parameters were varied in FSL. Eventually, the *polynomials order and the number of iterations* parameters were varied in BV

From the BranWeb simulator we also extracted the phantom volume, which is a simulated MR image representing an anatomical model of a healthy brain. The phantom volume is created by combining ten three-dimensional “fuzzy” tissue membership volumes: grey matter (GM), white matter (WM), cerebrospinal fluid (CSF), fat, muscle, skin, skull, glial matter, connective tissue, and background. In each tissue memberships volume, the value of each voxel represents the probability of the tissue to be found at that specific voxel. The MRI simulator combines the tissue membership volumes using weights estimated by Bloch equations (Kwan et al. 1999). These weights are assigned by the simulator depending on the pulse sequence parameters chosen, and can reproduce MR image contrast in a realistic manner (Collins et al. 1998; Kwan et al. 1999). For our study, we used default settings of simulator parameters to generate an INU- and noise-free T1-weighted image (Fig. 2a and d) in order to make our results comparable with previous studies on INU correction (Arnold et al. 2001; Ashburner and Friston 2005; Sled et al. 1998; Tustison et al. 2010; Vovk et al. 2005, 2006; Ying et al. 2009). The image was obtained using Spoiled Fast Low Angle Shot (SFLASH) pulse sequence, with TR = 18 ms, TE = 10 ms and 30° flip angle. The image space was 181 × 217 × 181 mm, with voxel sampling of 1 mm isotropic.

After obtaining the INU- and noise-free T1-weighted image from the MRI simulator, we multiplied it with the INU field image to generate an INU-corrupted T1-weighted image. Finally, we also added Rician-distributed noise to the INU-corrupted image. Noise levels were set at 1, 3, 5 and 7 % standard deviation compared to the intensity of the brightest tissue in the INU- and noise-free image (see Supplementary Figs. 1 and 2).

#### Method Settings

We examined the performance of the four INU correction methods with a wide range of input parameters. Specifically, we were interested in comparing the results obtained with the parameters giving the most accurate estimate among all combinations of examined parameters (enhanced configuration) and those produced using standard input parameters (default configuration). The INU correction methods were characterized by different input parameters, which will be described in the following sections.

FSL, FS and BV are conceived to work on brain-extracted images. Conversely, SPM does not require any brain extraction, as it uses built-in probability maps that delimit the region to be processed. To generate a standard brain mask to be used in FSL, FS and BV, we calculated the union of thresholded GM, WM and CSF probability images (*p* > 0.5), obtained from the MRI simulator. We also used the Brain Extraction Tool (BET) implemented in FSL (Smith 2002), to generate a set of brain masks with different extent, which was controlled by varying the fractional intensity parameter between 0.1 and 0.6. Values above 0.6 were excluded from the analysis due to heavy cortical erosion. In order to have comparable results, the masks were obtained running BET on the INU-free image and then applied to each method. The similarity of BET masks compared to the MRI simulator mask was assessed by quantifying the relative extent in terms of voxel number, as well its Dice similarity index (Zou et al. 2004).

##### Statistical Parametric Mapping

The INU correction method of SPM has two input parameters: the *regularization* and the *bias field smoothing*. By tuning the *regularization*, the method may be more or less sensitive to sharp transitions between image structures. Higher values tend to be more suited in the presence of smooth transitions whereas low regularization values make the method more sensitive to high frequency patterns. The default regularization factor in SPM is equal to 10^−4^. In our analyses on simulated data, we examine all the values implemented in the method: 0, 10^−5^, 10^−4^, 10^−3^, 10^−2^, 10^−1^, 1, 10. The *bias field smoothing* permits to model the smoothness of the INU field. The numerical value to be set is the cut-off of DCT bases expressed in mm. Only DCT bases of periods longer than the cut-off are used to describe intensity inhomogeneities. In the presence of a very smooth INU field, if the estimated INU field is not forced to be smooth, then it will demonstrate higher intensity variation due to different tissue types rather than pure intensity inhomogeneity artifacts. The default cut-off in SPM is equal to 60 mm. For our investigations, we varied the *bias field smoothing* between 30 and 150 mm, at 10 mm intervals.

##### FMRIB Software Library

The INU correction method in FSL allows multiple user-adjustable parameters. Among them, we selected the two parameters that, according to the developers (Zhang et al. 2001), have the largest impact on the imaging results: the *bias field smoothing* and the algorithm *iterations*. The *bias field smoothing* parameter controls the level of low-pass filtering applied to the estimated INU field. The numerical value to be set is the Full-Width Half-Maximum (FWHM) in mm, which is supposedly larger in case of larger INU smoothness. FAST assumes a default value of 20 mm. In our study, we varied the FWHM from 5 to 50 mm, at 5 mm intervals. The accuracy of the INU field estimate is also characterized by the number of times the intensity inhomogeneity correction algorithm is iterated. By default, FAST implements 4 iterations. We run the FSL method setting this parameter to 4, 8, 16 and 32 iterations.

##### FreeSurfer

N3, the method included in FS, permits the selection of several parameters. Nonetheless, according to the developers (Sled et al. 1998) and as stated in subsequent studies (Boyes et al. 2008; Zheng et al. 2009), two of them are crucial for the intensity inhomogeneity estimate: the *deconvolution kernel* and the *spline smoothing distance*. Accordingly, we focused our investigations on these two parameters. The *deconvolution kernel* controls the width of the probability distribution of the expected INU field, expressed in terms of FWHM. N3 uses a default value of 0.15. In our study, the *deconvolution kernel* was varied between 0.05 and 0.5, with intervals of 0.05. The smoothing approach implemented in N3 is based on the approximation of data by a linear combination of smooth basis functions, specifically B-splines. The smoothness is determined by the *spline smoothing distance* in mm, which refers to the distance between basis functions. The default value in N3 is 200 mm. Accordingly, we varied the *spline smoothing distance* from 50 to 300 mm at 50 mm intervals. We also set the *maximum number of iterations* to 1000 and the *stopping threshold* (the coefficient of variation in the ratio between subsequent field estimates) to 0.0001 to support accuracy over speed, as in previous studies (Boyes et al. 2008; Zheng et al. 2009).

##### BrainVoyager

The BV method requires the selection of two input parameters, *the polynomials order* and the number of *algorithm cycles* of INU correction, which have a major impact on the intensity inhomogeneity detection (Dawant et al. 1993; Hou et al. 2006). Low order values help to model slowly varying INU profiles, while high orders tend to better describe sharp variations in the intensity inhomogeneity. The default order of polynomials is set to 3 and the number of cycles to 2. We examined the effect of a *polynomials order* between 1 and 7 and a *number of cycles* between 2 and 5, following the recommendation of the developers (Dawant et al. 1993; Hou et al. 2006).

#### Performance Assessment

The performance of each algorithm was quantitatively evaluated on the estimated INU field, in line with previous studies (Arnold et al. 2001; Chua et al. 2009). To account for potential inconsistencies due to arbitrary scaling of the INU estimates, all the INU fields were normalized in intensity (Chua et al. 2009). Normalization was implemented by multiplying the estimated INU field by a scalar value *ω*, according to the formula by (Chua et al. 2009) as follows:

2 $$ \omega =\frac{{\displaystyle {\sum}_{i=1}^n\left({b}_{sim,i}\cdot {b}_{est,i}\right)}}{{\displaystyle {\sum}_{i=1}^n{\left({b}_{sim,i}\right)}^2}} $$

where *b*_*sim*_ and *b*_*est*_ are the simulated and the estimated INU fields, respectively, and *n* is the number of brain voxels.

The correspondence between the simulated and the estimated INU fields was then assessed by the root mean square error (RMSE) between the two images. The RMSE was defined as:

3 $$ RMSE=\sqrt{\frac{{\displaystyle {\sum}_{i=1}^n{\left(\omega {b}_{sim,i}-{b}_{est,i}\right)}^2}}{n}} $$

Being the RMSE a distance measure, the smallest RMSE indicated the best reconstruction performance. To complement the RMSE analysis, we also calculated spatial correlations between the simulated and estimated INU fields. Furthermore, the accuracy of the intensity inhomogeneity correction was assessed by visual inspection of the estimated INU fields and the relative histograms. Finally, we also estimated the effect of the INU correction on the actual images. To this end, we calculated the relative difference between the reconstructed and simulated T1-weighted images without INU contamination. The image *r*(*x*, *y*, *x*) representing the relative difference between the reconstructed image *ṽ*(*x*, *y*, *x*) and the simulated image *v*(*x*, *y*, *x*) is obtained by the following formula:

4 $$ r\left(x,y,x\right)=\frac{\tilde{v}\left(x,y,x\right)-v\left(x,y,x\right)}{v\left(x,y,x\right)} $$

As a representative value for a region of interest (ROI), we defined the Mean Absolute Relative Error (MARE) as the average of the absolute relative difference *r* across ROI voxels. Specifically, we evaluated the MARE value in the GM, WM, CSF, as well as in the whole brain, for different simulated INU fields and inhomogeneity correction methods.

## Results

Since INU correction algorithms are often used with standard input parameters, we initially examined the ability for each algorithm to detect intensity inhomogeneities according to the default and the enhanced configurations, respectively. To this end, we used the simulated T1-weighted image with INU 40 % and noise 3 % level. This analysis suggested that the default parameters do not always provide an estimate that is comparable to the one obtained using the parameters of the enhanced configuration (Fig. 2). For instance, the RMSE obtained with SPM with default settings was substantially larger than the minimal one for the BIAS 1.5 T profile (Fig. 2a), whereas for the BIAS 3 T and BIAS 7 T profile the difference was less pronounced (Fig. 2e and i ). In the case of BV, the RMSE values in the default configuration were almost double than the ones obtained with enhanced input parameters regardless of the INU field profile (Fig. 2d, h and l). For FS and FSL, the RMSE obtained with default configuration was similar to, but slightly larger than the one corresponding to the enhanced configuration (Fig. 2c, g and j and d, h and l, respectively). Furthermore, the analysis of the complete set of RMSE values obtained with each method suggested that the results obtained with SPM and BV are largely sensitive to the input parameters used, whereas FSL and FS provide relatively stable results.

**Fig. 3:**
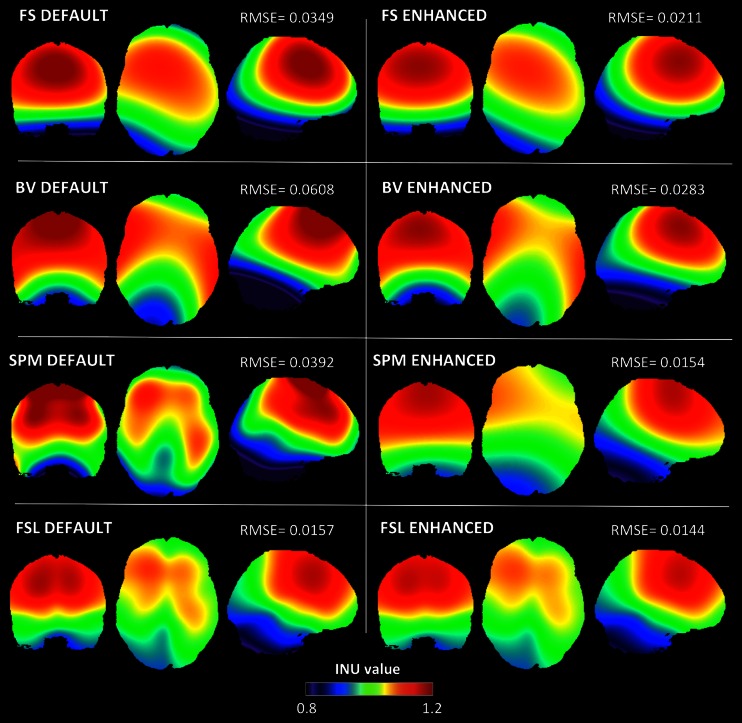
Estimated BIAS 1.5 T INU field for default and enhanced parameter configurations. BIAS 1.5 T INU field estimated by FS, BV, SPM and FSL with default (*left side*) and enhanced parameter configurations (*right side*), respectively. RMSEs between the simulated and the estimated INU field are also indicated

**Fig. 4:**
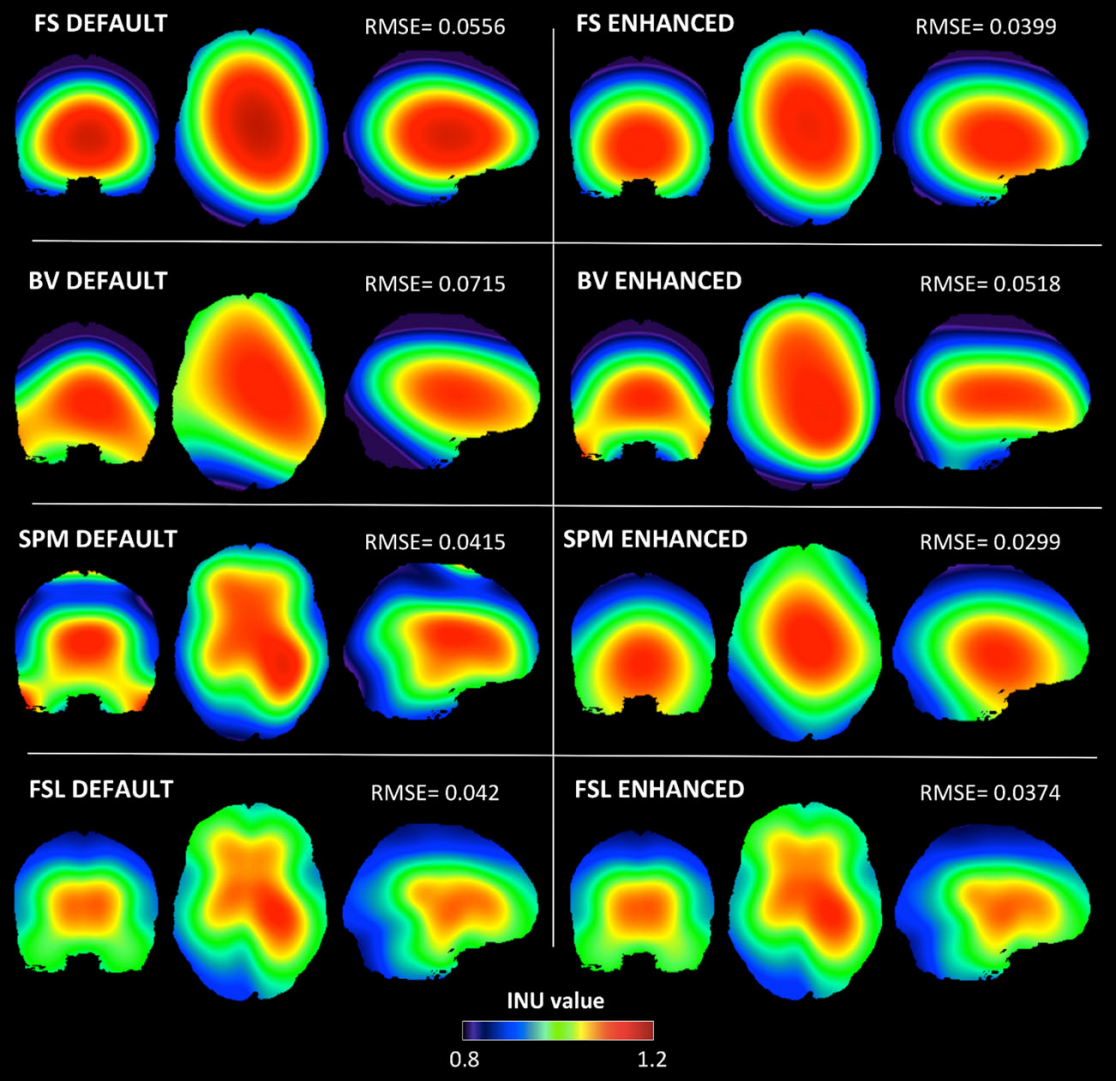
Estimated BIAS 3 T INU fields for default and enhanced parameter configurations. BIAS 3 T INU field estimated by FS, BV, SPM and FSL with default (*left side*) and enhanced parameter configurations (*right side*), respectively. RMSEs between the simulated and the estimated INU field are also indicated

**Fig. 5:**
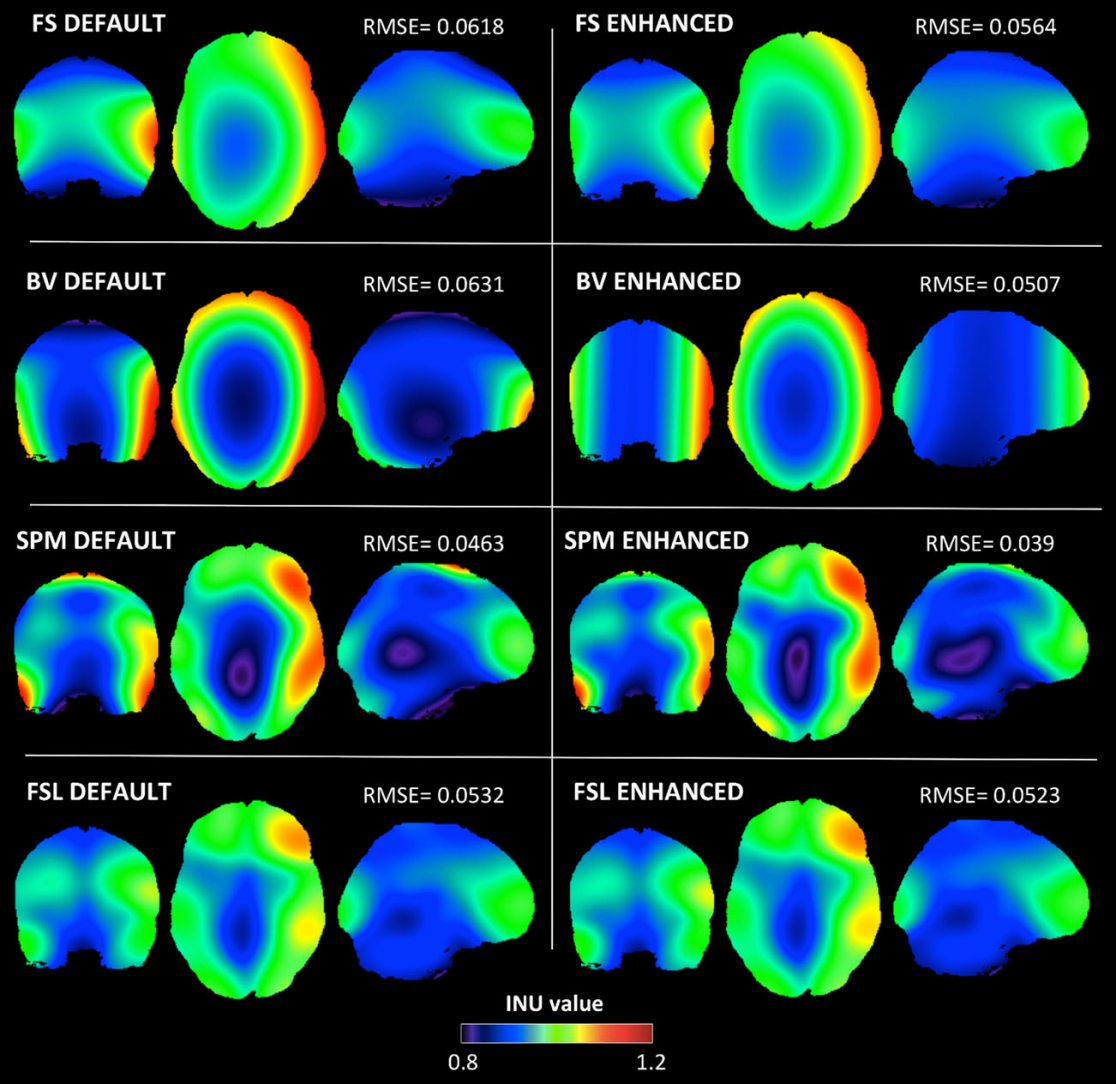
Estimated BIAS 7 T INU fields for default and enhanced parameter configurations. BIAS 7 T INU fields estimated by FS, BV, SPM and FSL with default (*left side*) and enhanced parameter configurations (*right side*), respectively. RMSEs between the simulated and the estimated INU field are also indicated

The quantitative results of the RMSE analysis were confirmed by a qualitative comparison of the INU fields produced by the four methods under their default and enhanced configurations. In the BIAS 1.5 T simulation (Fig. 3), we found that only FSL was able to reconstruct the INU field with relatively good accuracy when using default parameters. Much less accurate results were obtained with the other three methods, with BV showing the less reliable INU field estimate. When we examined the INU field maps obtained using the enhanced configurations, we found that the overall differences across algorithms were largely reduced. Among the four methods, BV was still characterized by a higher RMSE compared to FS, SPM and FSL. The spatial profile of the INU field provided by SPM substantially improved, along with the RMSE. When we selected input parameters based on the enhanced configuration, SPM and FSL converged to fairly similar INU field estimates. Also in the BIAS 3 T simulation (Fig. 4), SPM and FSL showed the best performance under default settings, while FS and BV were characterized by a less accurate reconstruction. When the enhanced configurations were selected, FS and FSL considerably improved their performance and showed similar results. Despite a slight improvement in the RMSE value, BV was still characterized by the poorest accuracy in the spatial profile. SPM showed the most accurate results, both in terms of RMSE and INU field profile. The high INU field complexity in the BIAS 7 T simulation revealed a generally less inaccurate reconstruction of the spatial profile both in the default and enhanced configuration (Fig. 5). When the enhanced parameter configuration was selected, all the four methods showed reduced RMSE values, with SPM yielding the smallest error.

**Fig. 6:**
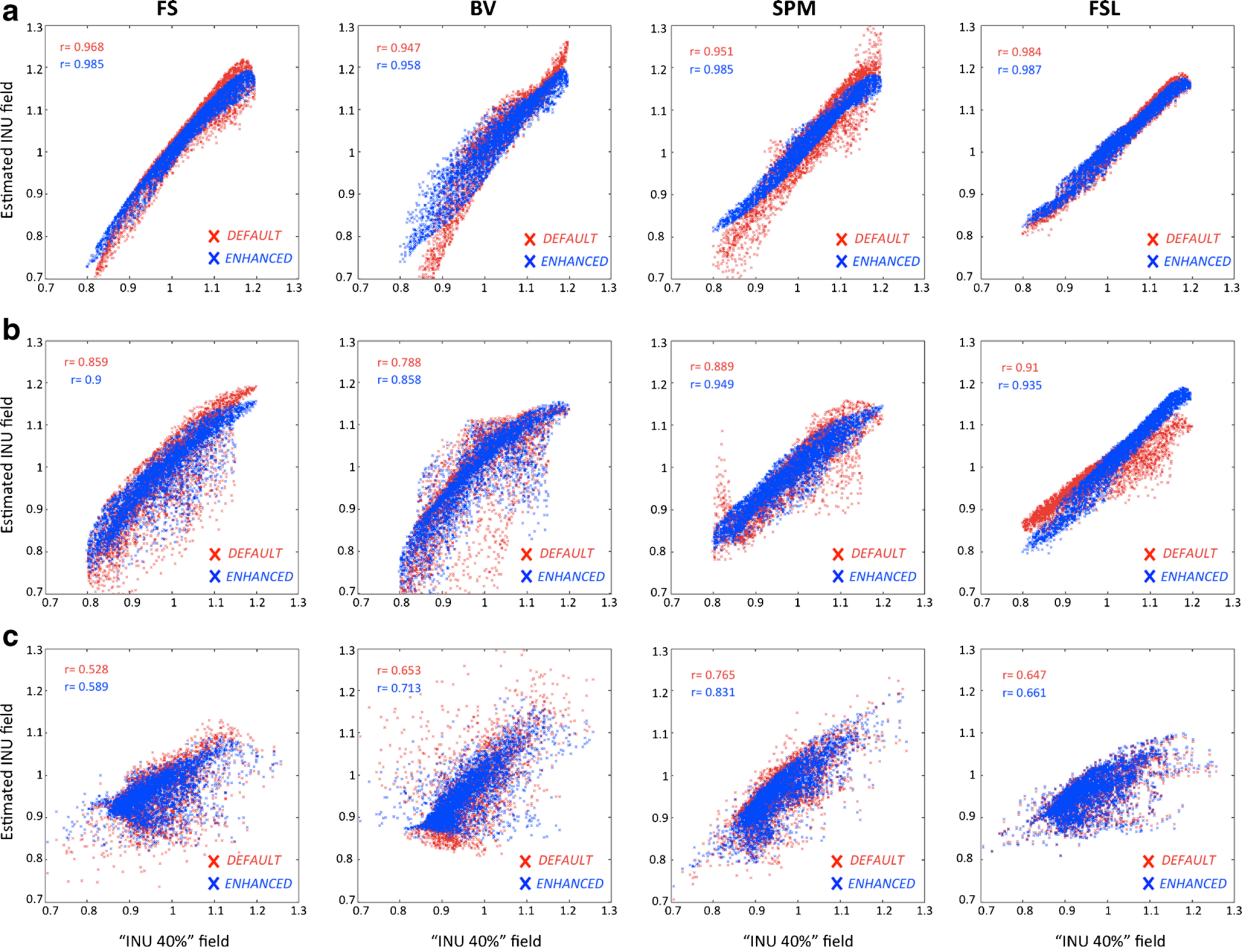
Scatterplot of INU fields obtained with default and enhanced parameter configurations. The scatterplot of the estimated INU field values for FS, BV, SPM and FSL with default and enhanced parameter configurations are illustrated in red and blue, respectively. The correspondence between the estimated and true INU fields was estimated using spatial correlations. The same analysis was performed for the BIAS 1.5 T (**a**), BIAS 3 T (**b**) and BIAS 7 T (**c**) simulations, respectively

A scatterplot analysis conducted on INU values also revealed significant improvements using enhanced parameter configurations (Fig. 6). Furthermore, the analysis of voxelwise correlations confirmed the results obtained using RMSEs in relation to the performance of the different INU correction methods. When we examined the histogram distributions of the estimated INU fields, we also observed that high RMSE (or low voxelwise correlation) values are not only explained in terms of a poor estimate in the spatial profile of the INU field, but also by an altered reconstruction of its values (Supplementary Fig. 3). Particularly, we noticed that FS, BV, and SPM under default configuration displayed INU values outside the range 0.8–1.2, which was the range of values of the simulated INU image. These broadened intensity distributions were still noticeable when considering enhanced configurations for FS and BV, but not for SPM and FSL.

It is also important to consider the effect produced by an imperfect INU field estimate on the actual image intensity. For this reason, we evaluated the difference between the simulated and reconstructed T1-weighted images, focusing on the results obtained by the methods with enhanced parameter configuration (Table 1). For this specific analysis and the subsequent ones, we focused on the enhanced parameter configuration of each method to exclude possible confounds associated with the choice of the default parameters. We obtained larger reconstruction errors for GM and CSF compared to WM, suggesting that the major discrepancy was at the outer edges of the brain, where the algorithms may tend to overcompensate/undercompensate the actual non-uniformity leading to spurious brightening/darkening. This finding was confirmed by close inspection of the relative error images. In both BIAS 1.5 T and BIAS 3 T simulations, FS and BV showed marked intensity variations between INU-corrected and INU-free T1-weighted. FSL and SPM were characterized by variations of modest magnitude across the whole volume, with the latter method showing generally smaller errors. Also the BIAS 7 T simulation showed SPM to be the method with the lowest reconstruction errors across the whole brain, in line with the analyses conducted using RMSE (Figs. 3 and 5) and spatial correlations (Fig. 6). With the complex INU profile of this simulation, BV provided a lower reconstruction error than SPM in WM, but higher in GM, CSF, and overall in the full brain.

**Table 1:** Image reconstruction error for each of the four INU correction methods

		WM	GM	CSF	FULL BRAIN
BIAS 1.5 T	FS	1.33	1.58	2.35	1.66
	BV	1.51	2.30	3.12	2.19
	SPM	1.02	1.24	1.44	1.20
	FSL	0.90	1.16	1.49	1.14
BIAS 3 T	FS	2.45	2.92	4.37	3.06
	BV	2.63	3.74	6.85	3.97
	SPM	2.28	2.50	2.81	2.48
	FSL	2.56	2.98	4.11	3.05
BIAS 7 T	FS	3.48	4.56	6.08	4.51
	BV	2.30	3.80	4.74	3.49
	SPM	2.86	3	4.33	3.22
	FSL	3.60	4.10	5.41	4.21

The mean absolute relative error (MARE) between the INU corrected T1-w image and the simulated T1-w image is shown for FS, BV, SPM and FSL with enhanced parameter configurations. The MARE was calculated for each of the three INU profiles using WM, GM, CSF and the full brain as regions of interest

**Fig. 7:**
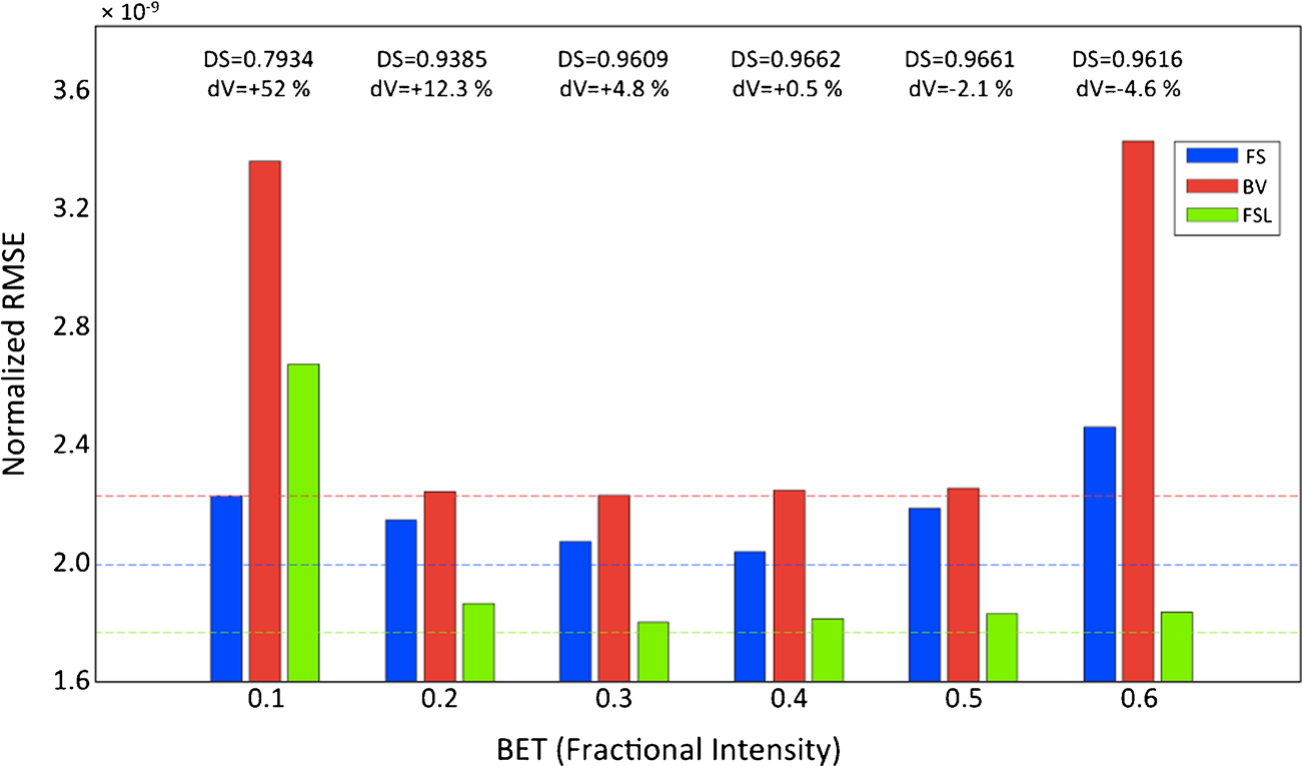
Dependence of the INU correction on brain masking. The impact of the brain extraction on the INU field estimate is shown for FS, BV and FSL only, since SPM does not need the specification of an explicit brain mask. Brain masking was performed using the Brain Extraction Tool (BET), using different *fractional intensity* values as input parameter. To allow the comparability of the results, the RMSE was calculated only for voxels in the intersection volume of the different masks obtained by BET. The RMSE values for INU estimates by FS, BV and FSL, with different fractional intensity as input for BET, are represented in a bar plot. Average values among the BIAS 1.5 T, BIAS 3 T and BIAS 7 T simulations are reported. *Dashed lines* represent the RMSE values for INU estimates using a standard mask (obtained from the MRI simulator), which are provided for comparison. Volumetric variations in the brain-extracted volume (dV) with respect to the standard mask are indicated over the bar plots, along with their Dice similarity (DS). The RMSE values are computed averaging the results of the three INU field profiles

Since FS, BV and FSL require a brain mask to be given as input, we evaluated the impact of brain extraction on the INU field reconstruction provided by each of these three methods. This analysis indicated that the definition of an image to be used as spatial mask had a relative impact on the INU correction performance (Fig. 7 and Supplementary Fig. 4). The performance of FS were dependent on the extent of the brain mask, as defined by the fractional intensity value set in the Brain Extraction Toolbox (BET). As expected, the most accurate INU correction with the FS method corresponded with the most accurate brain masking (fractional intensity value equal to 0.4), as indicated by the highest Dice Similarity value. A different result was found for BV, which yielded relatively stable RMSE values for intermediate extents of the brain mask (fractional intensity between 0.2 and 0.5). On the other hand, BV was characterized by high RMSE with the most conservative and the most extreme brain masking (fractional intensity equal to 0.1 and 0.6, respectively). Also FSL yielded relatively stable RMSE values, discounting the case of most conservative brain masking. Overall, the RMSE values obtained for FSL were substantially lower than for BV and FS.

**Fig. 8:**
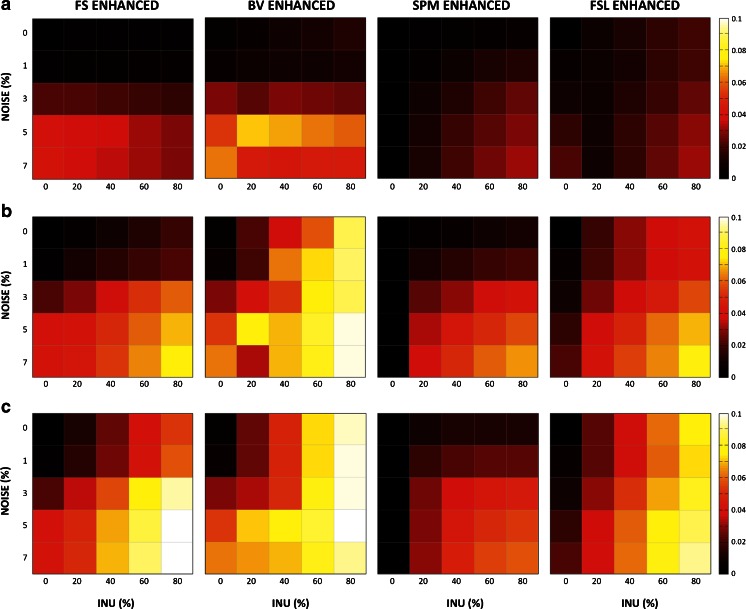
Sensitivity of INU correction methods to inhomogeneity magnitude and noise. FS, BV, SPM and FSL were compared in the enhanced parameter configurations for the BIAS 1.5 T (**a**), BIAS 3 T (**b**) and BIAS 7 T (**c**) profile. We calculated RMSEs between the simulated and the estimated INU fields for different INU field magnitudes and noise levels. The image with 40 % INU and 3 % noise levels corresponds to the one used for previous analyses

Finally, we evaluated the performance of the four methods for different levels of INU field magnitude and image noise (Fig. 8). This analysis revealed, as expected, an increased INU field magnitude and/or an increase image noise level generally yielded higher RMSE for all methods. Furthermore, all INU correction methods were more effective with a slowly varying INU field (BIAS 1.5 T) than one with complex profile (BIAS 7 T). SPM showed remarkable stability and accuracy for different noise levels and INU magnitudes, regardless of the INU field profile. FSL had comparable RMSE values to SPM for the BIAS 1.5 T field, irrespective of noise level and INU field complexity, whereas it was relatively less accurate with the BIAS 3 T and BIAS 7 T profiles. FS and BV generally underperformed the other two methods, and showed specific features related to the sensitivity to the two INU field and noise. Specifically, noise was the primary responsible for a reduction in the performance in both methods. Though, BV proved to be much more sensitive to the INU field magnitude than FS.

## Discussion

The correction of intensity non-uniformity in MR images is extremely important to ensure the reliability of investigations on brain structure. For instance, inadequate correction of the INU leads to decreased stability of automated segmentation algorithms (Clarke et al. 1995; Dawant et al. 1993; Pham and Prince 1999; Zheng et al. 2009) and this may in turn yield false positive and false negatives in voxel-based morphometry studies (Ashburner and Friston 2000; Good et al. 2001; Imabayashi et al. 2013). Here, we tackled the problem of assessing the performance of INU correction techniques, and in so doing we focused on the methods implemented in four widely used software for MR data analysis: FS, BV, SPM and FSL. Unlike several previous studies (Arnold et al. 2001; Boyes et al. 2008; Gispert et al. 2004; Zheng et al. 2009), we conducted our analyses on simulated MR images as this allowed us to assess INU field estimates against ground truth images. Our choice not to use actual MR images is due to the lack of validation studies and pending disputes regarding the reliability of performance evaluation measures (Chua et al. 2009). It should be considered, however, that simulated images may not account for scanner-specific variables and that representing the brain in terms of few tissue classes may not truly replicate the anatomical variation of a real brain. These potential limitations can be partly levied by adding random noise to simulated MR images, as we did in our study. Indeed, noise may be cautiously seen as “pseudo-anatomy” variations within tissues.

We believe that our study has provided valuable information with respect to four important aspects, which will be further discussed in the next sections. First, we found a large variability in the solutions provided by the INU correction algorithms. In particular, default parameter configurations did not always provide results sufficiently close to the results obtained with enhanced parameter configurations. Performance comparisons between different methods can be considered more objective if conducted using their enhanced configurations. Second, all methods under investigation are designed to remove slowly varying intensity inhomogeneities, and are relatively less effective with the more complex INU profiles that characterize high-field MR scanners. Third, the brain mask that needs to be given as input to FS, BV and FSL influences their performance. In contrast, SPM does not require the specification of any input mask. Fourth, approaches that integrate INU correction and brain segmentation, such as SPM and FSL, outperform methods dedicated to INU correction only, such as FS and BV, for different levels of INU magnitude and noise. Nonetheless, relatively accurate INU field reconstructions can be obtained with FS on MR images with low noise and with BV when the inhomogeneity magnitude is limited.

### Default and Enhanced Parameter Configurations

A large number of previous studies have compared the performance of several INU correction methods using default parameters (Arnold et al. 2001; Likar et al. 2001; Vovk et al. 2006). While those studies provided valuable information about the features of each method, it should be also considered that an objective comparison between methods could be best accomplished by using optimal input parameters. To get as close as possible to this condition, we examined a very large set of parameters and focused on the configuration with the relative minimal reconstruction error, which we denoted as enhanced parameter configuration. Notably, we carried out the comparison among INU correction methods on simulated data, since so far no approach to reliably measure INU correction performance exists for actual MR data (Chua et al. 2009). The identification of optimal set of input parameters for actual MR images would be extremely valuable, as it could increase the reliability of structural imaging analyses, and future work is warranted to develop an automated tool capable of performing such identification.

Among all algorithms under investigation, FSL was the only one that provided limited differences between the default and enhanced configurations. This suggests that the FSL method may be preferred in case of uncertainty about the selection of the adequate input parameters for INU correction. Conversely, we observed differences in RMSE values between default and enhanced configurations for SPM and FS, and partially also for BV. This can be explained by a substantial sensitivity of the INU reconstruction with respect to the input parameters used, as revealed by the analysis of RMSE values obtained with different combinations of input values (Fig. 2). For both SPM and FS, we also noticed that similar INU profiles could be estimated by simultaneously changing more than one input parameter. This can be the case when multiple parameters control the smoothness of the estimated INU. For instance, a similar RMSE could be obtained in SPM by increasing/reducing at the same time both the regularization and the smoothing factor (Fig. 2a, e and i). By close inspection of the RMSEs obtained with different combinations of input values (Fig. 2), we also noticed the presence of multiple local minima, which may be indicative of a complex pattern of interactions between the MR image to be corrected and the estimated INU field. This is not surprising, since specific MR image features may be detected as part of the intensity inhomogeneity depending on the algorithm parameters used.

It has been suggested that default input parameters for each INU correction method do not depend only on the method itself, but need to be identified also on the basis of the INU field profile, image quality (i.e. the signal-to-noise ratio) and the magnitude of the INU field (Arnold et al. 2001; Madabhushi and Udupa 2005). Zheng and coworkers highlighted the fact that most of the INU-correction methods were developed over a decade ago, and for this reason they are optimized to work well with low-field scanners only (Zheng et al. 2009). Likewise, other authors suggested specific input parameters should be properly selected to obtain accurate estimates (Boyes et al. 2008; Chua et al. 2009; Weiskopf et al. 2011). From this standpoint, the main elements to be considered are the strength of the static field and the geometry of the receiver coils (Boyes et al. 2008), as these influence also tissue-induced inhomogeneities. The current efforts put in the development of high-field MR scanners (Moser et al. 2012; Umutlu et al. 2014) suggest that parameter selection will become more relevant in the future, as the structural images will be more affected by the INU (Bernstein et al. 2006; Mihara et al. 2005; Uwano et al. 2014; Van De Moortele et al. 2005).

### Dependence of Correction Performance on INU Spatial Profile

It is a matter of fact that an increase of the static magnetic field has a significant impact on the spatial profile of the intensity inhomogeneity (Vaughan et al. 2001). Although several factors may influence the features of these non-anatomical variations, the magnetic field strength is the most important one (Belaroussi et al. 2006). MR images collected with 1.5 T scanners are characterized by a very smooth profile (Fig. 1a). On the other hand, localized intensity non-uniformity is observed in images acquired using 3 and 7 T magnetic fields (Fig. 1b and c), particularly in correspondence of outer brain structures as well as central regions. Furthermore, the spatial variations of the INU profile at 7 T are substantially larger then at 3 T (Bernstein et al. 2006; Collins et al. 2005; Umutlu et al. 2014). In this study, we considered three different INU profiles (referred to as BIAS 1.5 T, BIAS 3 T and BIAS 7 T), each representative of one of these three magnetic field strengths.

As we expected, all INU correction methods were substantially less effective with the BIAS 7 T profile compared to the BIAS 1.5 T and BIAS 3 T ones. When enhanced input configurations were considered, the SPM method proved to be the least affected by the fast spatial variations of the BIAS 7 T inhomogeneity. We argue that the use of a DCT basis functions may help to select the most important spatial frequencies for the INU reconstruction, and that this may be an important advantage of the SPM method compared to other ones. Notably, the FSL method provided accurate estimates with slowly varying INU fields, with performance largely similar to the SPM method (Figs. 3 and 4). On the other hand, the BIAS 7 T simulation clearly revealed a less accurate INU reconstruction with FSL compared to SPM (Fig. 5). A possible explanation may be in the different implementation of the bias field smoothing, which is not related to the use of DCT basis functions. As an alternative, reconstruction performance in FSL may be lower than in SPM, because FSL does not model the intensity distribution from different tissue types (e.g., GM, WM, CSF), which may be important to effectively discriminate fast spatial variations in the INU from normal intensity variations between brain tissues. As for the FS method, the input parameters that provided best performance were similar for the three INU profiles (Fig. 2b, f and j), suggesting a limited capability of adapting to an increased spatial complexity of the INU with higher magnetic field strength. This might be due to the fact that the deconvolution of narrow Gaussian distributions implemented in FS cannot easily discriminate the effects of noise and of high INU frequencies in the MR image. Similarly, BV showed a clear sensitivity to fast spatial variations that characterize the BIAS 7 T profile. The INU correction implemented in BV only relies on intensities of selected WM voxels (Hou et al. 2006). As such, the BV method may be less accurate when the spatial inhomogeneity does not vary slowly, as this condition evidently increases the uncertainty in the INU estimate far from the sampled WM voxels.

### Effect of Brain Extraction on INU Correction

Brain extraction is an important pre-processing step in brain imaging analysis. It is typically used before inhomogeneity correction, and may therefore influence its performance. We therefore assessed whether or not brain masking has an impact on the accuracy of INU reconstruction. This analysis was conducted on the methods implemented in FS, BV and FSL, since SPM requires no brain mask to be given as input. In line with our expectations, the brain extraction analysis revealed a substantial sensitivity of the INU field estimate with respect to the mask extent (Fig. 7). As for the BV method, we found very low RMSE variability using masks generated by BET with intermediate fractional intensity values. On the other hand, the poor accuracy shown with a conservative brain extraction (mask volume +52 % compared to that of the standard mask) as well as with a severe masking (mask volume −4.6 % compared to that of the standard mask) yielded to the conclusion that BV is particularly sensitive to the brain extraction process. A similar result was observed for the method implemented in FSL. In particular, the high RMSE value obtained with a very conservative masking suggested that brain extraction is fundamental to obtain a reliable INU correction. This is consistent with what suggested by the developers of FSL (Zhang et al. 2001). The FS method showed the highest dependence of the INU reconstruction for intermediate fractional intensity values, as compared to the other BV and FSL. Furthermore, our findings for FS are in agreement with those of Boyes and coworkers, who suggested that the inclusion of non-brain structures as well as parts of the background yields poor INU estimates (Boyes et al. 2008).

### Sensitivity to INU Magnitude and Noise Level

While we initially evaluated the methods at a single, realistic level of INU magnitude and noise, we then varied these two parameters to gain insights into the characteristics of each method. The two segmentation-based methods, that are the ones in SPM and FSL, proved generally superior to those in FS and BV (Fig. 8). This may be explained by the fact that the combination of INU correction and brain segmentation within the same framework yields advantages for both processing procedures (Ashburner and Friston 2005; Zhang et al. 2001). SPM and FSL can generally achieve good results for different noise levels and INU field magnitudes, with SPM being slightly more accurate for low levels of noise or magnitudes of the INU field. SPM makes use of information in template images for GM, WM and CSF. Essentially, these volumes encode the probability of finding different tissues at each spatial location. Notably, the use of tissue probability maps may have the favorable effect of modeling real intensity contrasts between different tissue types (Uwano et al. 2014). The INU correction approach of FSL, in turn, does not make use of prior information from a template and implements spatial encoding through a HMRF model. This means that also FSL uses contextual intensity information, but only with a spatial extent of few neighboring voxels. This approach may be particularly effective in the presence of intermediate levels of noise and INU field magnitudes. However, at very high levels of noise and INU field magnitude, FSL yielded larger RMSE values compared to SPM (Fig. 8b and c).

One of the main features that allowed N3, the method integrated in the FS pipeline, to become a method widely used by the neuroimaging community is its limited range of assumptions. It is indeed a histogram-based method that does not require any explicit model of the intensity distribution. Due to the intrinsic INU fields unpredictability, the need of no prior information on intensity properties is a noteworthy aspect. Intensity distributions in pathological cases are not known a priori, thus a model-independent assumption may be beneficial. According to our results (Fig. 8), N3 provides good estimates in presence of moderate INU field magnitude and noise levels, but is much less effective at higher values (i.e., 80 % INU level and 7 % noise). In heavily corrupted images, restoring high frequency patterns by deconvolving narrow Gaussian distributions from the MR image becomes trivial.

As for BV, the sensitivity to noise and INU magnitude becomes more important. The BV method is based on the idea that intensity variations within WM voxels may be used to extract INU information. Partial volume effects introduced by noise may hamper an accurate estimate of intensity inhomogeneity (Hou et al. 2006). Since this method can only make use of a limited subgroup of samples, the intensity normalization may be less accurate in areas where WM concentration decreases, as for example in the inferior part of the brain and the cerebellum (Figs. 3, 4 and 5). By the same token, high noise levels also affect BV performance. Since polynomial fitting is used to estimate the INU, the presence of noise may inevitably lead to a less accurate inhomogeneity correction.

## Conclusion

We have conducted a comprehensive assessment of INU correction methods for structural MR brain images. Specifically, we have generated structural images that accurately mimic those typically collected using 1.5, 3 and 7 T MR scanners, respectively. Accordingly, we have modeled intensity inhomogeneities with spatial profiles characterized by increasing complexity levels. It is worth noting that INU correction methods generally assume the intensity inhomogeneity to slowly vary across voxels. Such an assumption may not hold anymore with high-field scanners, for which the INU field variations are comparable to the dimension of the human brain structures (de Graaf et al. 2012). Our findings confirmed a generally worse INU correction for high-field MR images, suggesting that further work is warranted for the development of inhomogeneity correction methods that are effective also with complex INU profiles. Another important element to consider is that selection of valid input parameters of a given INU correction method may be better conducted by taking into account the characteristics of the MR image. Our findings provide a valuable basis for the selection of the INU correction method to be used. On the other hand, our study does not address the question of which input parameters should be used with actual MR data. Our future work will be focused on the development of a dedicated software tool capable of identifying, for any INU correction method, the parameter configuration that is the most appropriate for a given MR image. Such a tool might have a profound impact on the reliability of structural neuroimaging investigations.

## Information Sharing Statement

All simulated data and results of this work can be obtained by sending an email request to the corresponding author.

## Electronic supplementary material

ESM 1 (PDF 3152 kb)
