# Supplementary material for: Quantitative Evaluation of Intensity Inhomogeneity Correction Methods for Structural MR Brain Images
Source: Neuroinformatics. 2015 Aug 26;14:5–21. doi: 10.1007/s12021-015-9277-2 (PMC4706843; doi:10.1007/s12021-015-9277-2)
Supplement: Supplementary file 1 — (PDF 3152 kb) [file 12021_2015_9277_MOESM1_ESM.pdf]

Supplementary Material

Quantitative evaluation of intensity inhomogeneity correction methods for structural MR brain images

Marco Ganzetti[1,2], Nicole Wenderoth[1,3], Dante Mantini[1,2]

[1] *Neural Control of Movement Laboratory, ETH Zurich, 8057 Zurich, Switzerland;*

[2] *Department of Experimental Psychology, University of Oxford, Oxford OX1 3UD, United Kingdom;*

[3] *Laboratory of Movement Control and Neuroplasticity, KU Leuven, 3001 Leuven, Belgium.*

Corresponding author:

Dr. Dante Mantini

Department of Health Sciences and Technology, ETH Zurich

Winterthurerstrasse 190, 8057 Zurich, Switzerland

Telephone: +41 44 632 42 01

Fax: +41 44 632 42 56

Email: dante.mantini@hest.ethz.ch

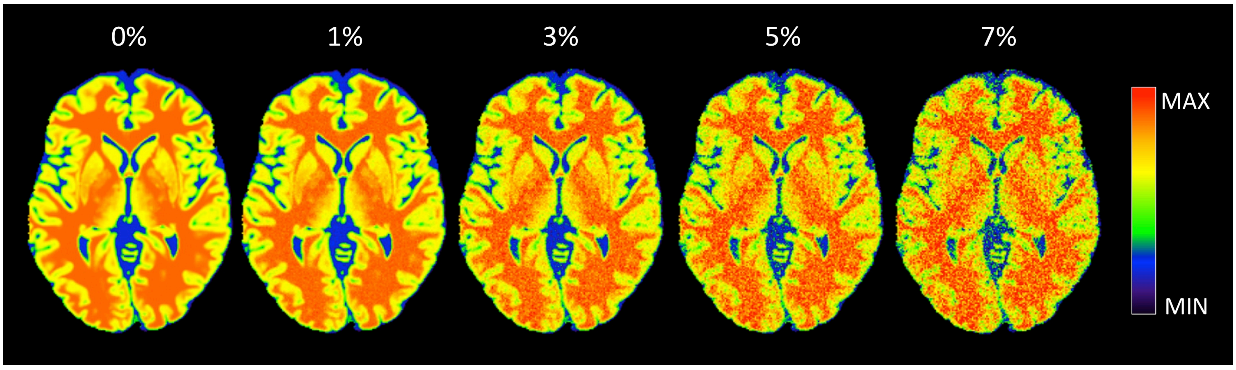

Supplementary Figure 1. Levels of Rician noise used in the simulations.

Noise with Rician probability distribution is superimposed on an INU-free T1-weighted image. Different noise levels were used in our simulations. The level (0%, 1%, 3%, 5%, 7%) is quantified as the standard deviation of the noise distribution relative to the brightest tissue of the INU-free image.

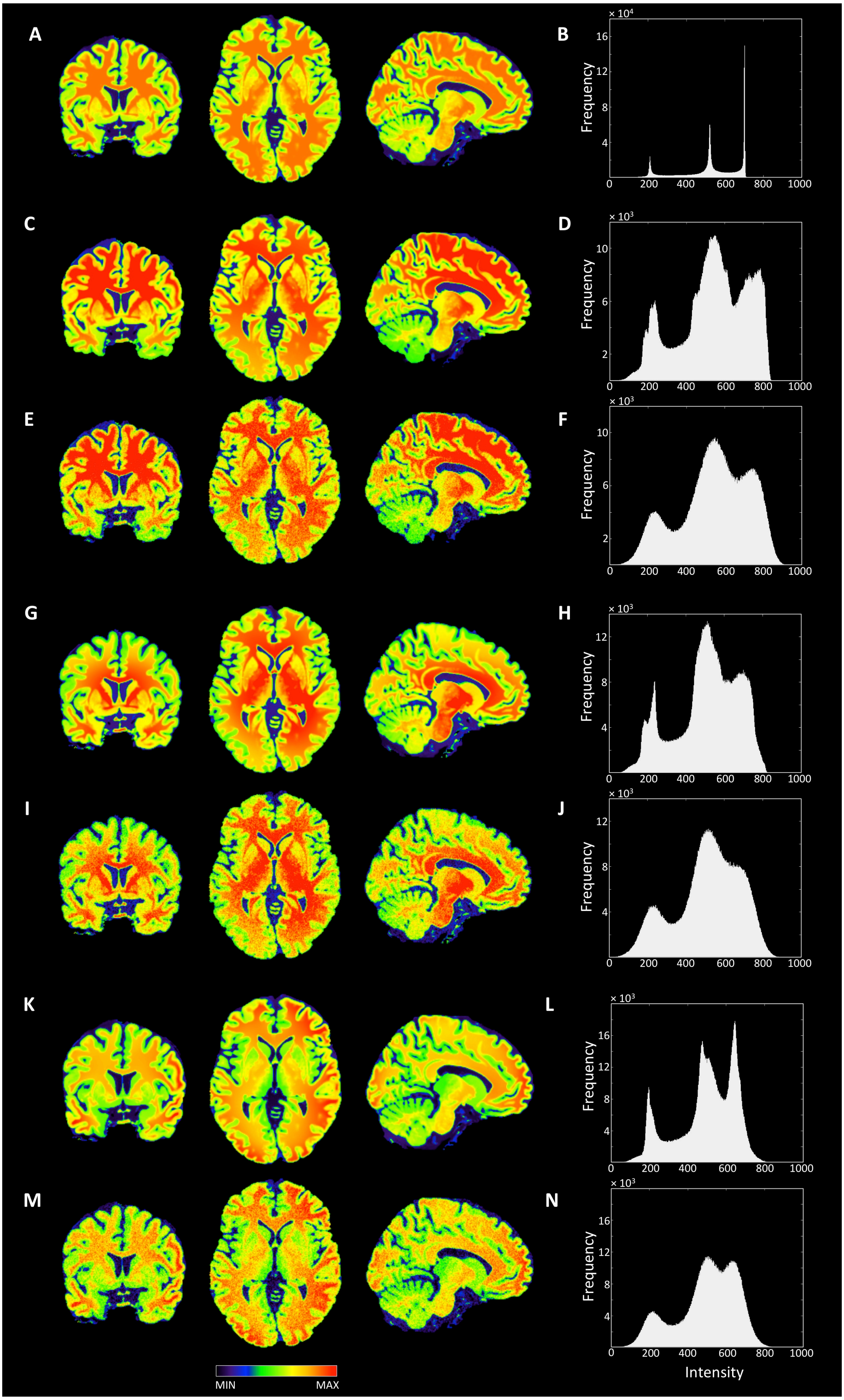
A
B
C
D
E
F
G
H
I
J
K
L
M
N
Frequency
Intensity
× 10⁴
× 10³
0 200 400 600 800 1000
MIN
MAX

Supplementary Figure 2. Modelling of MR intensity inhomogeneities.

The INU- and noise-free T1-w image (A) generated by the BrainWeb simulator is characterized by easily identifiable CSF, GM, and WM peaks in the intensity histogram (B). INU-corrupted T1-w images are generated by multiplying the INU- and noise-free T1-w image by the simulated BIAS 1.5T (C), BIAS 3T (G), BIAS 7T (K) fields respectively, at 40% level (with values ranging from 0.8 to 1.2). This leads to a broadening of CSF, grey matter, and white matter peaks in the respective intensity histograms (D,H,L). Rician noise at 3% relative intensity is added to the INU-corrupted T1-image to generate a INU- and noise-corrupted image (E,I,M). The histograms corresponding to this image (F,J,N) are smoother and have larger CSF, GM, and WM peaks.

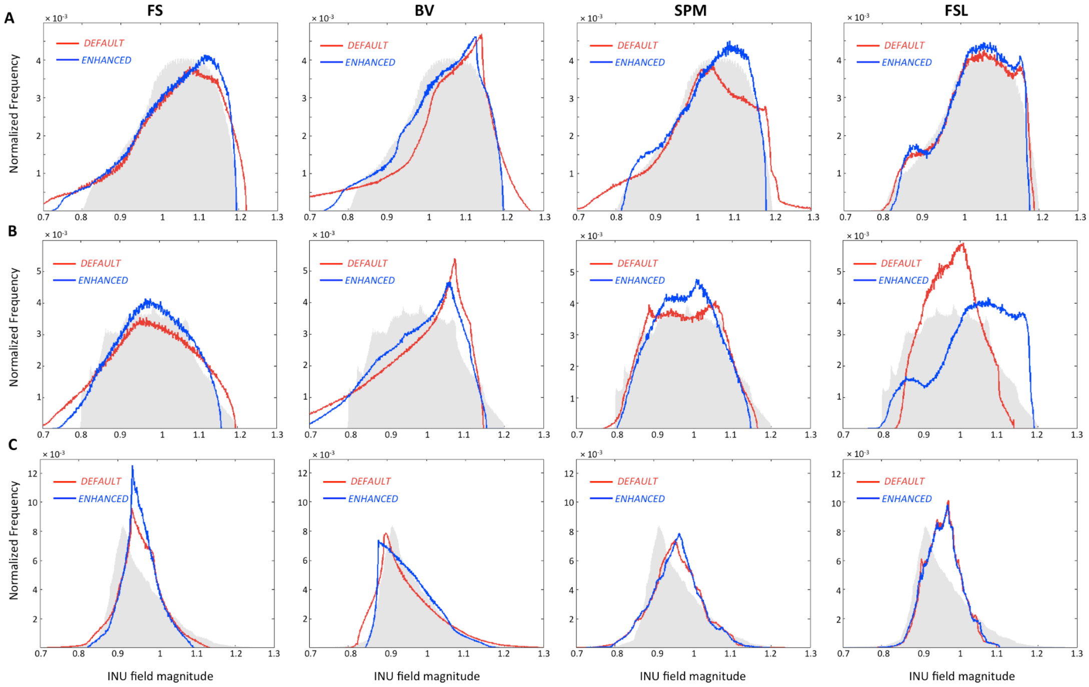

Supplementary Figure 3. Histogram of INU fields obtained with default and enhanced parameter configurations.

The histogram of estimated INU fields for FS, BV, SPM and FSL with default and enhanced parameter configurations are represented in red and blue, respectively. The histogram of the simulated INU field is also plotted for comparison in shaded grey. The same analysis was performed for the BIAS 1.5T (A), BIAS 3T (B) and BIAS 7T (C) simulations, respectively.

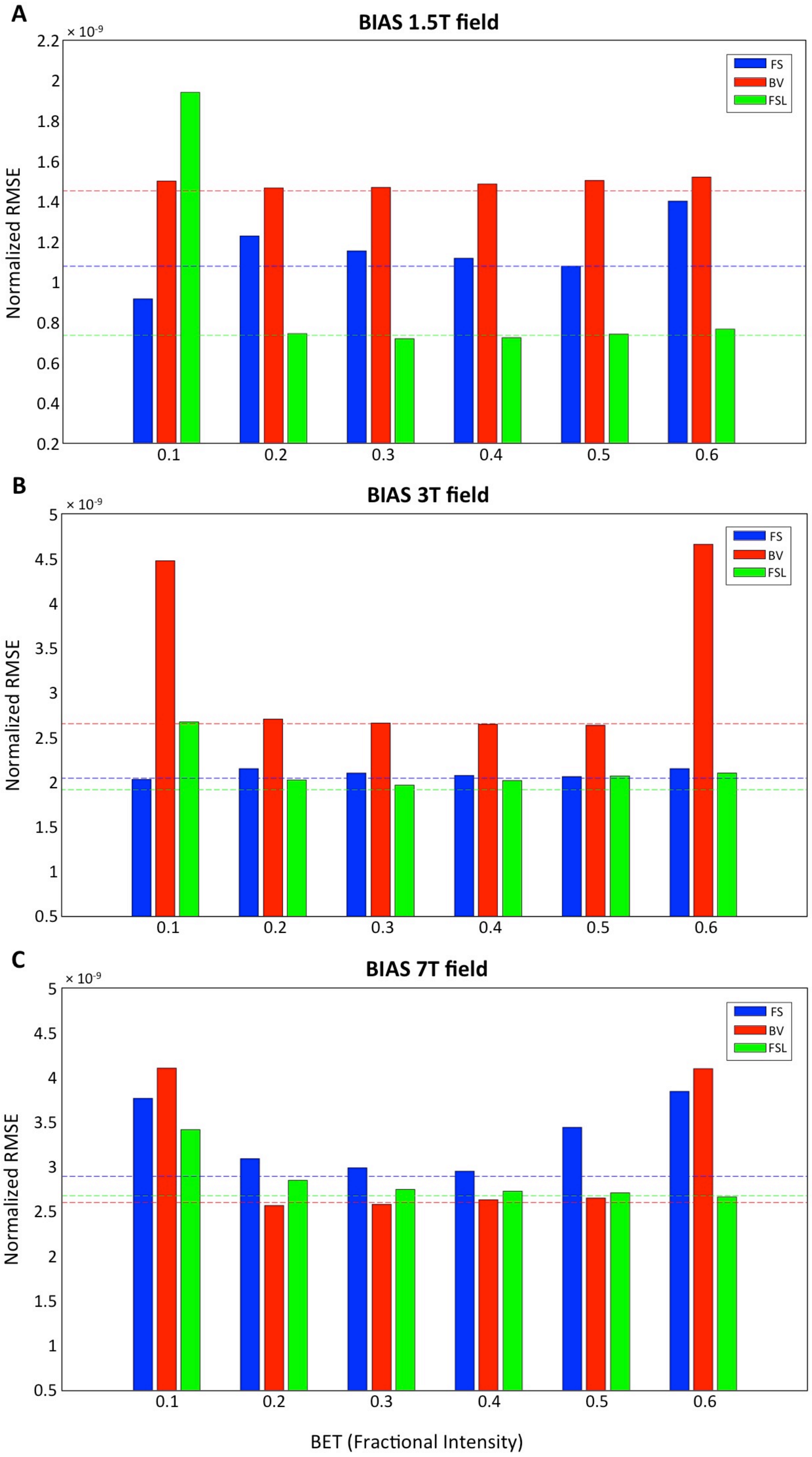
A
BIAS 1.5T field
× 10^-9
Normalized RMSE
FS
BV
FSL
0.1
0.2
0.3
0.4
0.5
0.6
B
BIAS 3T field
C
BIAS 7T field
BET (Fractional Intensity)

Supplementary Figure 4. Brain masking and INU correction.

We performed brain masking using the Brain Extraction Tool (BET), using different *fractional intensity* values as input parameter. We used the mask in FS, BV and FSL, which were assessed on the BIAS 1.5T (A), BIAS 3T (B) and BIAS 7T (C) datasets, respectively. To allow the comparability of the results, the RMSE was calculated only for voxels in the intersection volume of the different masks obtained by BET. Dashed lines represent the RMSE values for INU estimates using a standard mask, which are provided for comparison.
